# Genetic Diversity Analysis Reveals Genetic Differentiation and Strong Population Structure in *Calotropis* Plants

**DOI:** 10.1038/s41598-018-26275-x

**Published:** 2018-05-18

**Authors:** Nkatha G. Muriira, Alice Muchugi, Anmin Yu, Jianchu Xu, Aizhong Liu

**Affiliations:** 1 Key Laboratory for Economic Plants and Biotechnology, Yunnan, Key Laboratory for Wild Plant Resources, Kunming Institute of Botany, Chinese Academy of Sciences, Lanhei Road 132, Heilongtan, Kunming, 650201 Yunnan China; 2 University of Chinese Academy of Sciences, Beijing, 100049 China; 3 World Agroforestry Centre, East and Central Asia Office, 132 Lanhei Road, Kunming, 650201 Yunnan China; 4 World Agroforestry Centre (ICRAF), P.O. Box 30677-00100, Nairobi, Kenya; 5 Key Laboratory for Forest Resources Conservation and Utilization in the Southwest Mountains of China, Ministry of Education, Southwest Forestry University, Kunming, 650224 China

## Abstract

The genus *Calotropis* (Asclepiadaceae) is comprised of two species, *C*. *gigantea* and *C*. *procera*, which both show significant economic potential for use of their seed fibers in the textile industry, and of their bioactive compounds as new medicinal resources. The available wild-sourced germplasm contains limited genetic information that restricts further germplasm exploration for the purposes of domestication. We here developed twenty novel EST-SSR markers and applied them to assess genetic diversity, population structure and differentiation within *Calotropis*. The polymorphic information index of these markers ranged from 0.102 to 0.800; indicating that they are highly informative. Moderate genetic diversity was revealed in both species, with no difference between species in the amount of genetic diversity. Population structure analysis suggested five main genetic groups (K = 5) and relatively high genetic differentiation (*F*_ST_ = 0.528) between the two species. Mantel test analysis showed strong correlation between geographical and genetic distance in *C*. *procera* (r = 0.875, p = 0.020) while *C*. *gigantea* showed no such correlation (r = 0.390, p = 0.210). This study provides novel insights into the genetic diversity and population structure of *Calotropis*, which will promote further resource utilization and the development of genetic improvement strategies for *Calotropis*.

## Introduction

The genus *Calotropis* (Asclepiadaceae), which is native to the tropics and subtropics of Africa and Asia, has great potential for use as a fiber and medicinal plant^1,2^. Its long, fine seed hair (similar to that of cotton) is a high quality fiber, while the sap contains unique chemical compounds that have the diverse bioactive and pharmacological properties indicative of potential for new drug discovery^3^. In addition, *Calotropis*’ growth properties include drought hardiness, fast growth after establishment, a short reproductive cycle and adaptation to salty soils. This suggests that it is an important shrub which could serve as a key plant for ecological restoration in arid and semiarid regions^4,5^.

There are two closely related species of the genus; *Calotropis procera* (mainly distributed in the tropic and subtropics of Africa) and *Calotropis gigantea* (mainly found in tropic and subtropics of Asia). Due to their morphological similarity, with major differences occurring only on the floral structures, distinguishing them during the non-flowering season is difficult^4^. Taxonomically, *C*. *procera* is distinguished from *C*. *gigantea* by its flower characteristics such as subglobose flower buds, corona as long as gynostegium and erect corolla with five petals with dark purple tips^4^. Development of a genetic marker to distinguish the two species would facilitate species identification outside the flowering season.

Utilization of *Calotropis* products have mostly been explored from wild populations, which raises concerns regarding their sustainability and low product yield. Currently, there is a *Calotropis* domestication trial as a fiber source underway in Kenya (personal communication). The available wild germplasm has limited genetic information, with few molecular markers pinpointed that separate the two species. This currently limits germplasm exploration and genetic improvement of *Calotropis*. To date, various marker systems have been used in population genetic analysis of *Calotropis*. In flower polymorphic (pink and white) *C*. *gigantea*, genetic analysis using random amplified polymorphic DNA markers (RAPDs) found rich genetic diversity and high genetic similarity^6^. In 18 accessions of *C*. *procera* studied in Egypt, RAPDs unveiled a high polymorphism level^7^. Moreover, RAPDs were also used to discriminate thirteen salt tolerant plant species including *C*. *procera* according to their genetic relationships^8^. Analysis of three populations of *C*. *procera* on the basis of their climo-geographic adaptations using zymograms of superoxide dismutase and peroxidase indicated high genetic variation^9^. Further, SDS-PAGE for protein and isozyme in genetic variation analysis successfully discriminated between the genotypes of the studied *C*. *procera* populations^10^. The analysis of *C*. *procera* from Benin using Amplified Fragment Length Polymorphism (AFLP) uncovered high genetic variation among the sampled populations^11^.

However, these previous studies were based on only a few samples within a limited sampling area and mainly focused on *C*. *procera*. In addition, AFLP and RAPDs are dominant markers which cannot distinguish between heterozygotes and homozygotes, thus their use may over-estimate genetic diversity, making them less robust for use in genetic analysis^12^. On the other hand, isozymes are phenotypic markers whose usefulness is limted by the requirement for fresh samples, the fact that they are influenced by environmental changes and that only a few loci are analysed^13^. Expressed sequence tags-simple sequence repeats (EST-SSR) are more robust and efficient in population genetic analysis based on their intrinsic characteristics such as codominance, multi-allelic, highly reproducible, transferability across taxon, hypervariable and ubiquitous distribution across the genome^14^. In particular, EST-SSRs target protein coded are conservative and their markers are usually transferable within species, thus they are often applied to investigate the genetic diversity and population structure across related species^15–18^.

**Figure 1:**
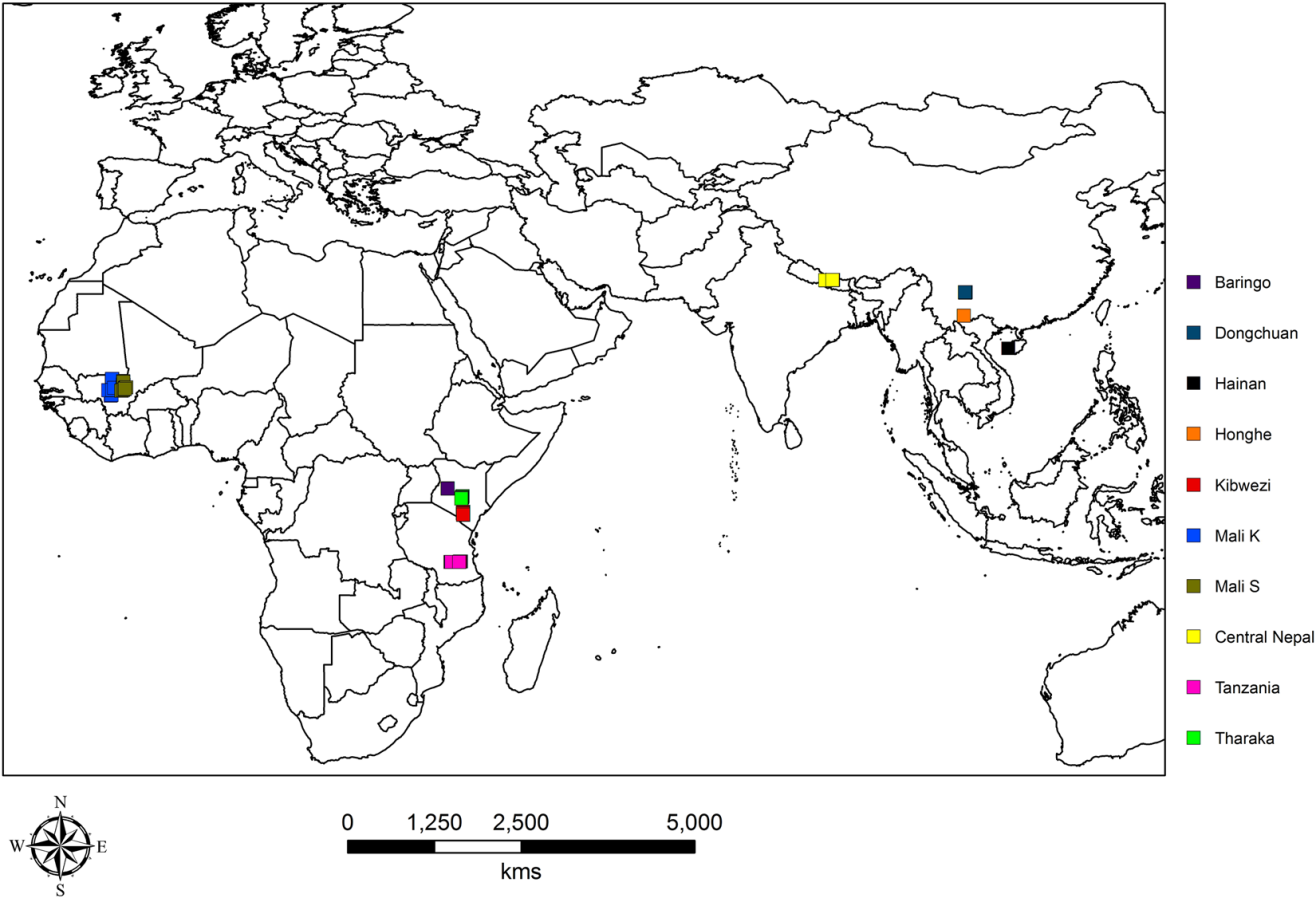
Map showing geographical locations where samples were collected. The marked points are exact points of collection. ArcGIS v10.2.2 (http://www.esri.com/) was used to generate the map.

In our previous study^19^, we generated transcriptome data for *C*. *gigantea*. Using that data, in the present study, we developed 20 polymorphic and efficient EST-SSR markers. These markers were used to investigate genetic diversity and differentiation and perform population structure analysis for ten populations collected from Asia and Africa (as shown in Fig. 1). These results provide novel insights into the genetic diversity and population differentiation in *Calotropis*. This study not only increases our understanding of the genetics of *Calotropis*, but also is useful to development of feasible strategies for *Calotropis* genetic resource management and conservation.

## Results

### Polymorphism of EST-SSR Markers

In total, 170 primer pairs were randomly selected from those designed from the transcriptome of *C*. *gigantea* in our recent study^19^. To inspect polymorphism of primers the screening was initially carried out using eight samples (four from *C*. *gigantea* and four from *C*. *procera*), selected at random from the sampled populations. Among the 170 selected primer pairs, 151 amplified successfully across *C*. *gigantea* and *C*. *procera* while 19 pair primers did not yield any PCR products at diverse annealing temperatures. Out of the 151 successful primer pairs, 109 yielded amplification products of the expected size, and the other 42 primer pairs generated PCR products that were larger or smaller than expected or unspecific bands. Of those successfully amplified, 20 primers (13.2%) showed polymorphisms whereas 89 were identified as monomorphic. The 20 polymorphic EST-SSR primers (see Table S1) were used for further population analysis. In total, 97 alleles were identified from *C*. *gigantea* samples, ranging from two (CG71) to eight (CG 84), with an average of 4.85 alleles per locus, whereas in *C*. *procera* samples, 84 alleles were identified with an average of 4.2 alleles per locus (Table S2). Across the20 loci, observed heterozygosity (*H*_O_) had a mean of 0.223 in *C*. *procera* and 0.194 in *C*. *gigantea* while the mean genetic diversity (*H*_S_) was 0.487 and 0.379 for *C*. *gigantea* and *C*. *procera* respectively. *PIC* values ranged from 0.065 to 0.769, with the mean = 0.429 in *C*. *gigantea* and 0.045 to 0.670, with the mean = 0.338 in *C*. *procera* (Table S1). From the average number of alleles and PIC values, it is evident that both *C*. *procera* and *C*. *gigantea* accessions display similar amount of genetic diversity (Table S1). Combining all populations of both species; the *PIC* ranged from 0.102 (CG71) to 0.800 (CG28). Inbreeding coefficient (*F*_IS_) values ranged from −0.365 to 0.879 (mean = 0.167) in *C*. *gigantea*, and from −0.597 to 0.951 (mean = 0.177) in *C*. *procera* (Table S2).

### Population Genetic Diversity Analysis

The genetic diversity (*H*_S_) of the ten populations ranged from 0.157 to 0.363 with an average of 0.245. In *C*. *gigantea* the average *H*_S_ was 0.249. The Nepal population showed the highest (*H*_S_ = 0.363), while the Dongchuan population had the lowest genetic diversity values (*H*_S_ = 0.157). In *C*. *procera* the Mali S population had the highest genetic diversity (*H*_S_ = 0.339) and the lowest was the Kibwezi population (*H*_S_ = 0.195) with a mean of 0.248. The average levels of observed heterozygosity (*H*_O_) across all populations was *H*_O_ = 0.214 while in *C*. *procera* and *C*. *gigantea H*_O_ was 0.223 and 0.198 respectively, while within species the level of genetic diversity (*H*_S_) was 0.249 and 0.256 for *C*. *gigantea* and *C*. *procera*, respectively. Allelic richness (*A*_R_) ranged from 2.25 (Honghe) to 3.3 (Nepal) in *C*. *gigantea* and 2.05 (Kibwezi) to 2.85 (Mali S) in *C*. *procera*. The fixation index (*F*) for all the ten populations ranged from 0.048 (Kibwezi) to 0.320 (Honghe). Using various genetic diversity parameters there was no noticeable difference between the two species (Table 1).

**Table 1:** Summary statistics: genetic diversity estimate at 20 EST-SSR loci for *C*. *gigantea* and *C*. *procera*. *N*, sample size; *A*_R_-Average allelic richness; *H*_O_-Observed Heterozygosity; *H*_S_-genetic diversity; *F*-fixation index.

Population/Species	N	AR	HO	HS	F
***C***. ***gigantea***					
Dongchuan	30	2.6	0.13	0.157	0.23
Honghe	30	2.25	0.152	0.194	0.32
Nepal	25	3.3	0.292	0.363	0.205
Hainan	30	2.4	0.22	0.25	0.192
Total/Mean	115	2.64	0.198	0.249	0.234
***C***. ***procera***					
Baringo	28	2.15	0.211	0.196	0.139
Tharaka	30	2.45	0.187	0.199	0.098
Kibwezi	29	2.05	0.185	0.195	0.048
Tanzania	30	2.35	0.227	0.282	0.145
Mali K	27	2.55	0.244	0.278	0.18
Mali S	27	2.85	0.288	0.339	0.098
Total/Mean	171	2.4	0.223	0.248	0.117

### Population Genetic Structure

**Figure 2:**
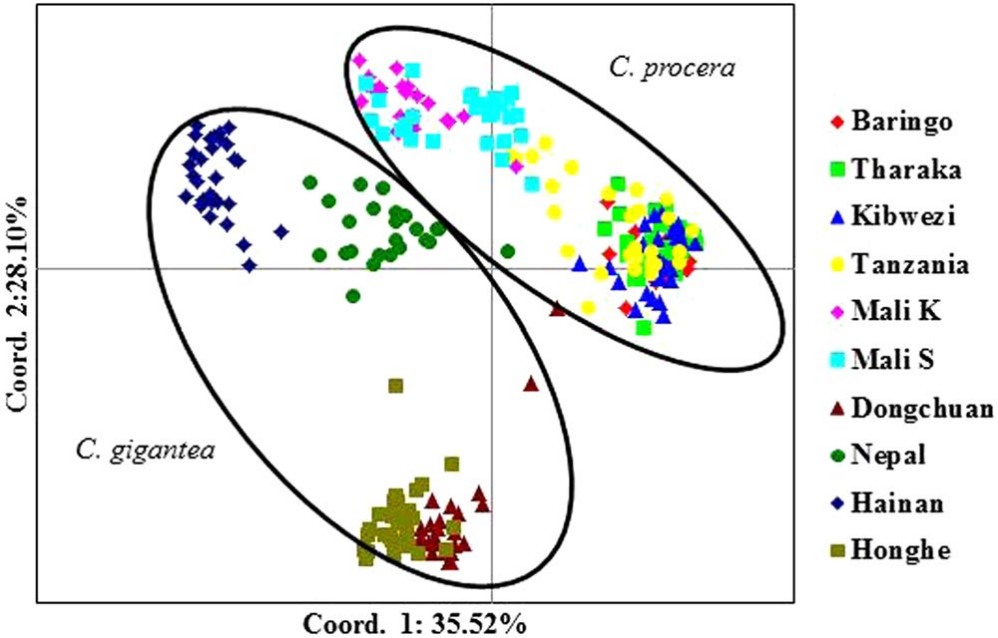
Scatter plot of two axes from a PCoA of 286 Genus *Calotropis*, explaining 64.62% of the total observed variation.

**Figure 3:**
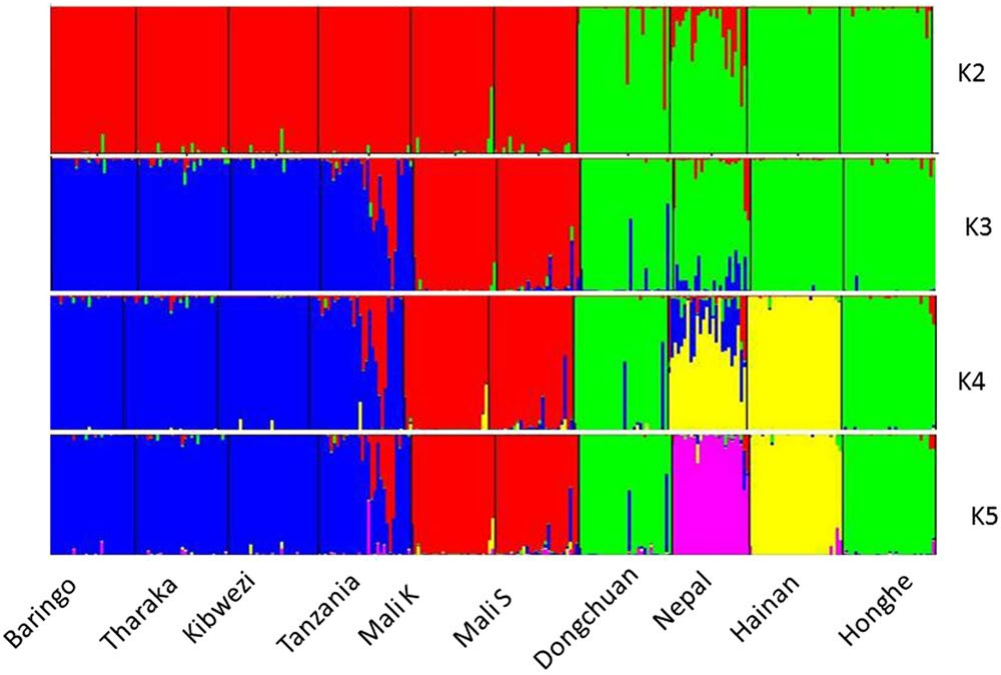
Bayesian STRUCTURE bar plot based on probabilities for 286 individuals of 10 populations of *Calotropis*. Black lines separate populations.

**Figure 4:**
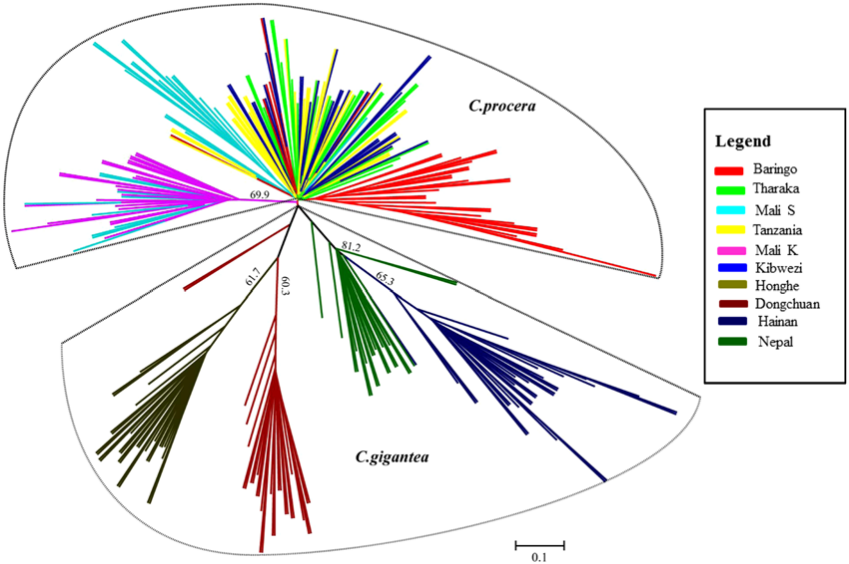
Radial Neighbour joining (NJ) tree showing relationships among populations of *Calotropis*. Bootstrap numbers (>60) were denoted on the lines.

PCoA is often used to show genetic similarity among populations, with populations clustered according to their geographical location and species identity. Our PCoA results showed that the two species were clearly clustered and that the first two axes explained 64.62% of the total observed variation, suggesting that a distinct genetic structure exist between *C*. *gigantea* and *C*. *procera* (Fig. 2). STRUCTURE analysis indicated the optimal cluster number as K = 5 based on delta K (Fig. S1). Due to their biological relevance, other genetic structure K = 2, K = 3 and K = 4 are also displayed in Fig. 3. At K = 2, strong genetic structure was found among our samples that corresponded to respective species. Further at K = 5, the results revealed that Nepal, Mali S and Tanzania populations each had a unique gene pool (see Fig. 3). In addition, we performed the genetic relationship analysis among populations with neighbor joining criteria. As shown in Fig. 4, the sampled individuals were clearly grouped into two clusters (*C*. *gigantea* and *C*. *procera*), in concordance with the results of PCoA and STRUCTURE analyses.

**Figure 5:**
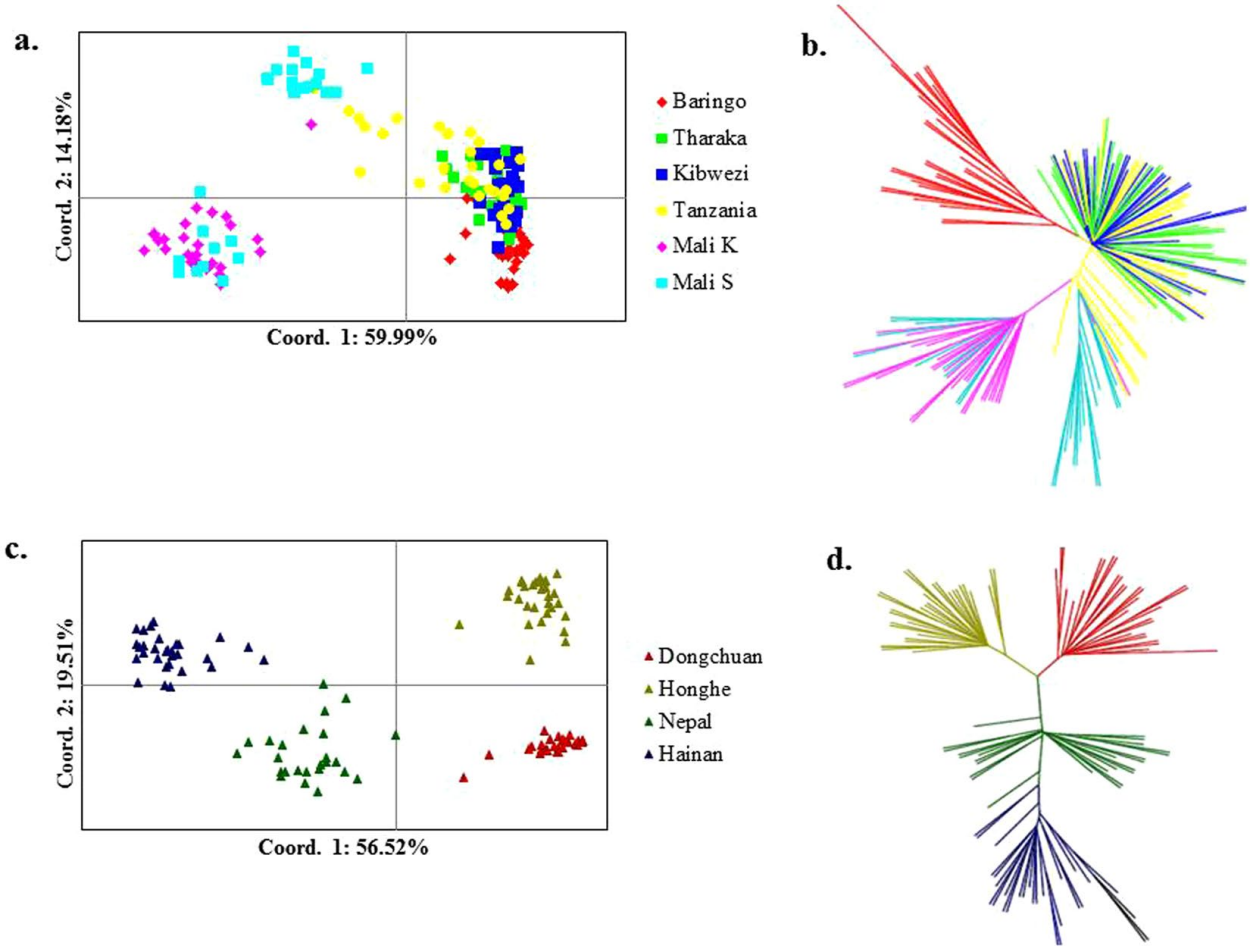
Principal coordinate analysis (PCoA) and Neighbour-joining (NJ) tree showing the relationships among populations of *C*. *procera* and *C*. *gigantea*. (**a**) PCoA for six populations of *C*. *procera*. (**b**) NJ for six populations of *C*. *procera*. (**c**) PCoA for four populations of *C*. *gigantea*. (**d**) NJ for four populations of *C*. *gigantea*. In each case, the colours correspond to the populations in NJ and PCoA.

**Figure 6:**
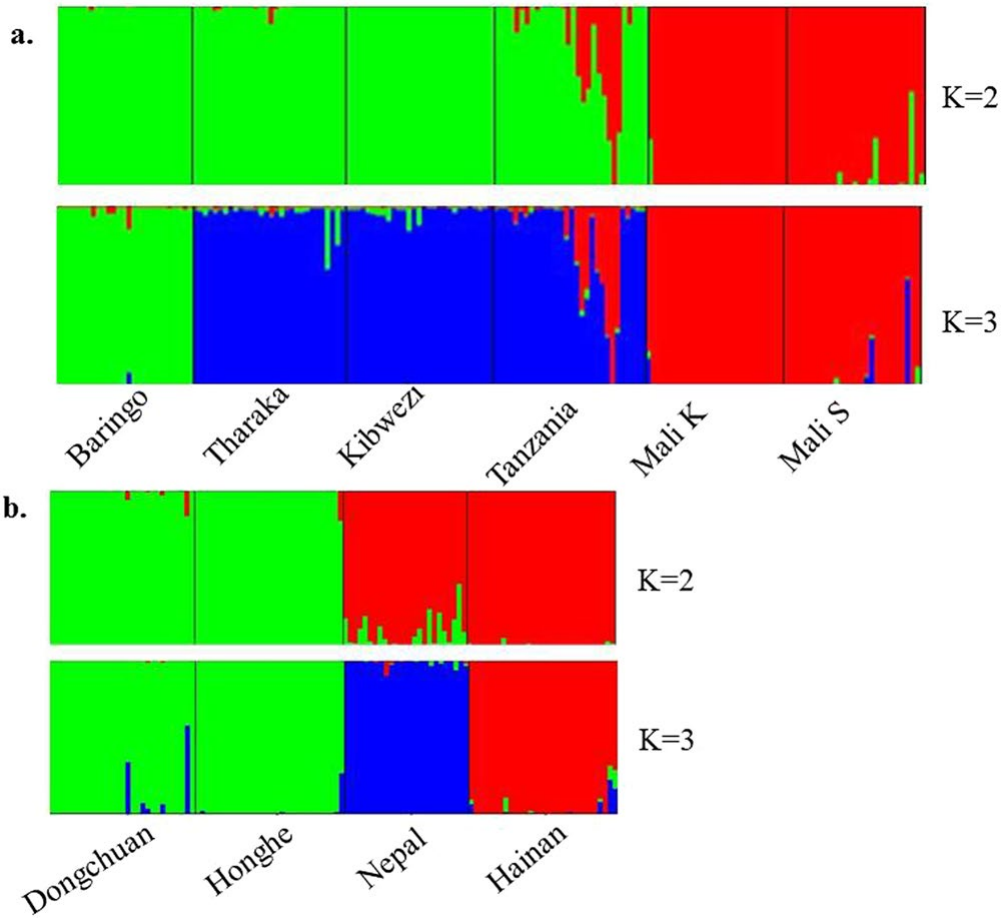
Structure bar plots showing the assignment of individuals into distinct genetic clusters. (**a**) *C*. *procera* (**b**) *C*. *gigantea*.

Intraspecies population structure analysis gave the optimum K value as K = 2 and K = 3 using Delta K and L (K) approaches respectively. Since delta K may erroneously result into K = 2 (Figs S2 and S3) both K Values were considered. In *C*. *procera* at K = 2, the populations grouped into West Africa (Mali K and Mali S) and East Africa populations (Baringo, Tharaka, Kibwezi, Tanzania). At K = 3 Baringo population separates from the rest of the East Africa populations, with Tanzania and Mali S populations showing admixtures (see Fig. 5). In *C*. *gigantea* populations at K = 2, the Dongchuan population clustered with the Honghe population while the Hainan population grouped with the Nepal population, however at K = 3 the Nepal population and the Hainan population each formed a separate group (Fig. 5). These results were consistent with the analyses from both PCoA and NJ (Fig. 6).

### Genetic Differentiation

Patterns of genetic divergence by AMOVA analysis showed that 28.32% of total variation was accounted for by interspecific differences between *C*. *procera* and *C*. *gigantea*, with a *F*_ST_ value of 0.528. When AMOVA analysis was assessed within each species, *C*. *procera* had most genetic variation (55.35%) partitioned within individuals while in *C*. *gigantea* among-population divergence was highest (57.01%). The genetic differentiation (*F*_ST_) for *C*. *procera* and *C*. *gigantea* were 0.366 and 0.57, respectively (Table 2). Among the sampled populations interspecies pairwise *F*_ST_ values were lowest between the Tharaka and Kibwezi populations both within *C*. *procera* (*F*_ST_ = 0.200) and the highest differentiation (*F*_ST_ = 0.491) was between the Baringo (*C*. *procera*) and Hainan (*C*. *gigantea*) populations (Table S3). Intraspecies pairwise *F*_ST_ values in *C*. *procera* showed that the most differentiated populations were between Baringo and Mali K (*F*_ST_ = 0.362), while in *C*. *gigantea* the Dongchuan and Hainan populations were the most differentiated (*F*_ST_ = 0.451) (Table S3). At the loci level the genetic differentiation (*F*_ST_) ranged from 0.036 (CG71) to 0.707 (CG83) with a mean of 0.405 in *C*. *gigantea* whereas in *C*. *procera* the *F*_ST_ ranged from 0.024 (CG35) to 0.764 (CG83) with a mean of 0.291 (Table S2).

**Table 2:** AMOVA analysis comparing genetic variation within and between species of genus *Calotropis* (*p* = value: 0.001).

Source of variation	Degrees of freedom	Sums of squares	Variance components	Percentage variation	Fixation indices
**(a)** ***Calotropis procera*** **vs** ***Calotropis gigantea***					
Between species	1	464.919	1.66740 Va	28.32	F_ST_:0.528
Among populations within species	284	1796.873	2.10756 Vb	35.8	F_IT_:0.601
Within populations	286	604	2.11189 Vc	35.87	F_IS_:0.156
**(b) Within** ***C***. ***procera***					
Among populations	5	434.203	1.47379 Va	36.66	F_ST_:0.367
Among individuals within populations	165	473.14	0.32118 Vb	7.99	F_IT_:0.447
Within individuals	171	380.5	2.22515 Vc	55.35	F_IS_:0.126
**(c) Within** ***C***. ***gigantea***					
Among populations	3	564.74	3.22907 Va	57.01	F_ST_:0.570
Among individuals within populations	111	324.79	0.49128 Vb	8.67	F_IT_: 0.658
Within individuals	115	223.5	1.94348 Vc	34.31	F_IS_:0.203

**Figure 7:**
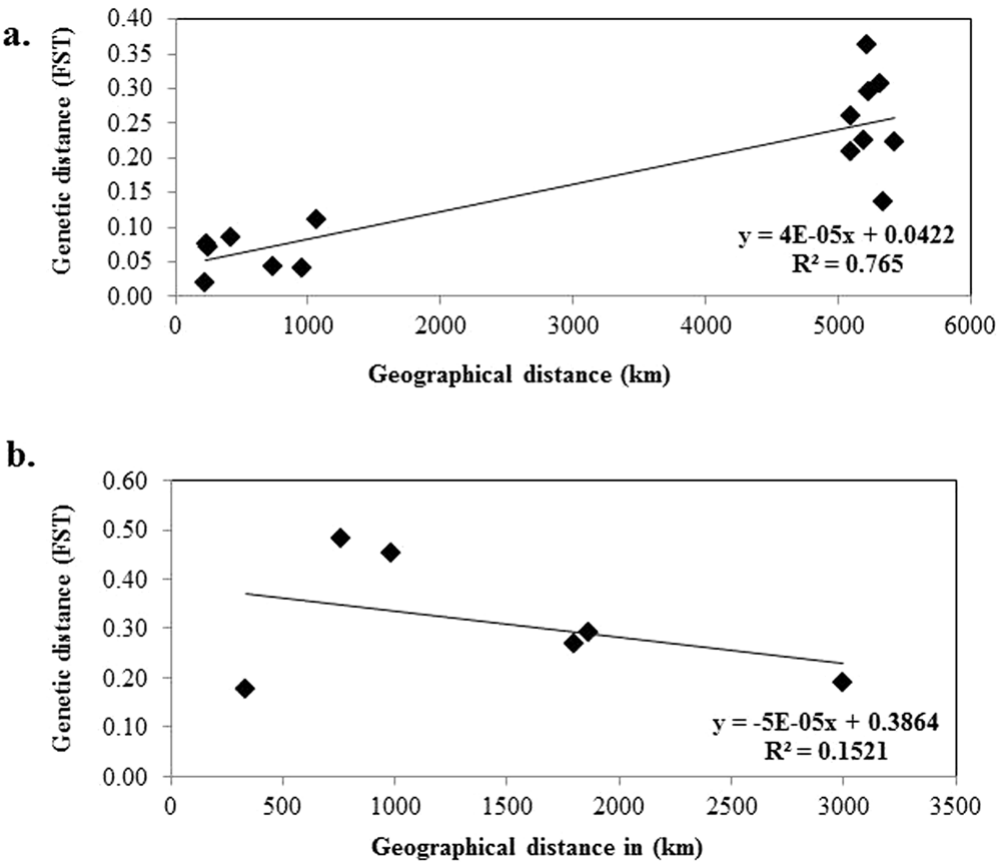
Correlation of geographic distance (in kilometers) and genetic distance (pairwise *F*_ST_) among 286 individuals of 10 populations of *Calotropis*, including regression line (Mantel test, R2 = 0.31, P = 0.001 at 1000 randomization).

Mantel test analysis is often used to examine the correlation between geographic and genetic distance^18^. Our Mantel test analysis showed a significant correlation between geographic and genetic distance among *C*. *procera* populations (r = 0.875, p = 0.020) (Fig. 7a), whereas no such correlation was found within *C*. *gigantea* populations (r = 0.39, p = 0.21) (Fig. 7b).

## Discussion

### Development of EST-SSR

This study represents the first attempt to develop and utilize EST-SSR markers to examine genetic diversity in the genus *Calotropis*. EST-SSRs have been found to be important molecular markers for detecting genetic diversity, structure, and demography as well for applied and experimental research on plant populations^20^. These markers show high transferability to closely related species of same genus or even family since they are associated with transcribed genes conserved among homologous genes^21^. Here, 151 primer pairs (88.8%) were successfully amplified PCR fragments from 170 pairs that had been designed from unigenes generated from transcriptome data. 19 primer pairs did not yield any PCR product. This could have been a result of insertions, lack of specificity, assembly errors, chimeric primers and the presence of large introns^22^. The polymorphism rate of *Calotropis* EST-SSR primers was 13.5%, which is comparable to that reported from other plants such as *Neotropteri nidus* (11%)^22^ and sesame 11.9%^23^.

The informativeness level of markers based on *PIC* is usually defined as low (*PIC* < 0.25), medium (0.5 > *PIC* > 0.25) or high (*PIC* > 0.5)^24^. Based on this criteria, the EST-SSR markers developed for *Calotropis* have a moderate level of polymorphism, with seven having high polymorphism, 12 having medium polymorphism and only one having a low *PIC* value (Table S1). The slight difference in *PIC* between *C*. *procera* and *C*. *gigantea* may have simple been due to bias introduce by deriving the markers from *C*. *gigantea*. This is congruent with what was observed in *Tilia platyphyllos* and *Tilia cordata*, in which the former (from which the markers were designed) had a slightly higher genetic diversity^17^. Overall, the developed EST-SSR markers showed sufficient polymorphism levels across the loci to perform further analyses of genetic diversity, differentiation and population genetic structure in *Calotropis*. This could contribute to future *Calotropis* genetics and breeding research, in areas such as identification of elite germplasm and marker-assisted selection.

### Genetic diversity analysis

It is crucial to assess genetic diversity in order to ensure that the most diverse populations are selected to widen the genetic base of germplasm. Based on the developed EST-SSR markers the current study assessed genetic diversity in both *C*. *procera* and *C*. *gigantea*. Genetic variation was lower than in previous genetic studies of *Calotropis* populations^9–11^. However, these studies are not directly comparable since different marker systems could result in slight differences in the results obtained.

Generally, species with a wide distribution, wind dispersal and outcrossing show high genetic diversity^25^, however, based on EST-SSR markers, *Calotropis* displays a moderate genetic diversity (*H*_S_ = 0.245). This level is comparable to the genetic diversity in *Blighia sapida*, a woody perennial species widespread in tropics and subtropics which had an average genetic diversity of *H*_S_ = 0.29^26^. It seems that the genetic diversity of *Calotropis* plants is lower than that of most outcrossing species^25^. The population genetic diversity did not differ between *C*. *procera* (*H*_S_ = 0.248) and *C*. *gigantea* (*H*_S_ = 0.249), despite the fact that *C*. *procera* was distributed widely in Afica and *C*. *gigantea* was fragmentally distributed in Asia. Generally, in fragmented populations genetic variability is expected to be low^27^, however, this might not be universal, and in *C*. *gigantea* there was no evidence of low variability. The European Beech has fragmented populations that nonetheless show no evidence of loss of genetic variability^28^. Within *C*. *procera*, the Mali S populations had the highest genetic diversity while in Nepal, *C*. *gigantea* had the highest genetic diversity (Table 1). These two populations could be an important source of germplasm for incorporating in future breeding programs.

### Population genetic structure

A plant population’s genetic structure is determined by the interaction of processes such as geneflow, mutation, selection and mating strategy^29^. The PCoA, STRUCTURE and NJ results clearly demonstrated genetic differentiation between *C*. *procera* and *C*. *gigantea*. Intraspecies structure analysis within *C*. *procera* and *C*. *gigantea* suggested that the most likely number of populations was K = 3, which was clustered primarily according to geographical region. Within *C*. *procera* the Baringo population split from the rest of East Africa population; this could be explained by limited geneflow between the populations as a result of extensive distance between them. Within *C*. *gigantea*, distinct groups were present as shown by both PCoA and NJ. Overall, the Nepal, Mali S and Tanzania populations had unique populations with admixed genotypes likely to harbor novel and potentially beneficial alleles. Thus these populations should be prioritized as source of germplasm for breeding and planting during domestication. Both interspecies and intraspecies analysis showed that a strong genetic structure existed in *Calotropis*, thus efforts into collection of germplasm should focus on sampling the maximum number of populations, to maintain high levels of genetic diversity^30^.

### Genetic differentiation

Although *C*. *procera* and *C*. *gigantea* are morphologically close, AMOVA revealed high between-species genetic differentiation (*F*_ST_) 0.528 (Table 2). In *C*. *gigantea*, the highest partitioning of genetic variation was found among populations. Such partitioning is expected for species with mixed mating systems^25^ rather than for those with outcrossing systems such as is suggested to be the probable mating system in *Calotropis*. This could be as a result of the sampled populations of *C*. *gigantea* occurring within fragments due to natural barriers such as mountainous terrain. This restricts the movement of insect pollinators to far distances, resulting in pollination occurring only within clumps of close relatives. These results are comparable to those of *Hippophae tibetana* Schlect^31^, and *Cycas simplicipinna*^32^ populations, which were found to have high genetic differentiation as a result of barriers limiting gene flow. However, partitioning of genetic variance in *C*. *procera* found high within-population variation and lower *F*_ST_ than in *C*. *gigantea*. This is an indication of relatively unrestricted geneflow between most of the sampled populations, resulting in a low genetic differentiation because *C*. *procera* is continuously distributed in African regions. Mantel test analysis found a strong correlation between geographical and genetic distance in *C*. *procera* (r = 0.875, p = 0.020). Populations separated by greater distances were more genetically dissimilar than those populations that are geographically close - which led to stronger internal genetic differentiation. Therefore, it is likely that the genetic structure of this species is affected by geographical distance. This high isolation by distance implies that selection and use of genetically diverse genotypes are key factors in *C*. *procera* breeding program during domestication and development of varieties with a broad genetic base. However, genetic isolation by distance is not a static process and may change with time, resulting in changes in genetic composition of a given population. In *C*. *gigantea*, however, Mantel test showed no correlation between geographic distance and genetic distance pattern (r = 0.39, p = 0.21), which further supports our hypothesis that geographical isolation exists among studied populations of *C*. *gigantea*, which led to genetic high differentiation which is suggested to have resulted from population fragmentation, low gene flow and genetic drift, similar to that in white Jabon (*Anthocephalus cadamba*)^33^.

## Conclusion

We developed the first EST–SSR markers, thus providing a strong impetus for genetic analysis in *Calotropis* spp. Although the EST-SSR markers were of moderate polymorphism they showed power in discriminating between two closely related *Calotropis* species. These markers are also linked to functional genes due to their location in the coding regions of the genome; thus, may be useful for functional analysis of traits of interest. We found moderate genetic diversity between the two species of *Calotropis* with no difference within them. Information on genetic diversity will ensure that the maximum genetic diversity can be captured during the domestication process. We found strong interspecies and intraspecies genetic structure in this genus, thus collection of germplasm efforts should focus on sampling the maximum number of populations in order to preserve a high level of genetic diversity. These markers of genetic differentiation will give insights into incorporating the most diverse populations into breeding programs. Overall this study will be a useful resource for further genetic research in *Calotropis*.

## Materials and Methods

### Sample Collection and DNA Extraction

We sampled 286 individuals of *Calotropis* from 10 natural populations from Africa and Asia, at a minimum distance of 50 meters. From each sampling point individuals’ GPS readings were taken which were then transformed into reference points and mapped (Table S4 and Fig. 1). Two young leaf samples were collected in replicates, then immediately put in silica gel to dry until DNA extraction. Total DNA was isolated following cetyltrimethyl ammonium bromide (CTAB) method^34^. The DNA integrity and quality was measured by Nanodrop 2000 spectrophotometer (Thermofischer scientific, wilmington, DE, USA) and also by running on 1.0% (w/v) agarose gel. The DNA for PCR amplification was then diluted to a final concentration of 50 ng/µL for each sample in TE buffer (10 mmol/L Tris-HCL, PH 8.0, 1 mmol/L EDTA).

### SSR Marker Screening

We selected 170 primers pairs at random from those designed in our recent study^16^. The EST-SSR markers were screened for PCR amplification using eight individual samples selected at random, four samples from Asia (*C*. *gigantea*) and four from Africa (*C*. *procera*). Polymerase chain reactions (PCR) were performed in 12.5 µL reaction volumes containing 6.25 µL 2X easytaqPCR PAGE MasterMix (TransGen Biotech, Beijing, China), 0.5 µL of forward and reverse primers, 0.5 µL of genomic DNA (50 ng/µL) and ddH2O 4.75 µL. Amplification of the PCR products was carried out using a BIO-RAD T100TM Thermal cycler (Singapore) with the following cycle: initial denaturation at 95 °C for 5 min; 35 cycles of 30 s at 94 °C, 45 s at a range of annealing temperatures to attain the optimum (Table S1); 30 s of elongation at 72 °C, and a final extension for 10 min at 72 °C. The PCR products were then separated by electrophoresis at 180 V and 50 W on 8% non-denaturing polyacryramide gel and visualized by silver nitrate staining^35^. The polymorphic primers were then selected and utilized in genotyping 286 samples.

### EST-SSR Genotyping

Polymerase chain reaction (PCR) amplification of the 286 *Calotropis* samples using 20 developed loci was carried out in a 20 µL volume. The total volume contained 10X PCR buffer (2 µL), 25 mM MgCl2 (1.6 µL), 10 mM of dNTPs (0.4 µL), 5 unit/µL of Taq DNA polymerase (0.15 µL) TaKaRaTaqTM kit (TAKARA BIO INC., Dalian, China), 10 pmol each of forward and reverse primers (0.4 µL), 40–50 ng/µL of DNA template (1 µL) and 14 µL of sterile ddH2O. The forward primers of all the selected primers were fluorescent labeled with a 6-FAM, HEX and TAMRA (GENEray Biotech, Shanghai, China). PCR amplification conditions were as follows: 5 min at 95 °C, 35 cycles of 30 s at 94 °C, 45 s annealing temperatures 54 °C–62 °C (Table S1), 30 s at 72 °C and 10 min 72 °C final extension.

The success of amplification was determined by running the PCR products on 1% agarose in 1xTAE buffer. After successful selective amplification 1 µL of PCR product was mixed with 0.5 µL of size standard GeneScanTM 500 LIZ (Applied Biosystems) and 9 µL HI-DI^TM^ (Applied Biosystems), denatured and then separated on an ABI 3730xl Genetic Analyzer (Applied Biosystems, USA).

### Data Analysis

The SSR allele sizes was estimated by Genemarker v4.0 (Softgenetics LLC, State College, PA, USA) for all populations, checked manually and entered in a spreadsheet. We used PowerMarker v3.25^36^ to calculate *PIC*, *A*_R_, number of alleles (*N*_A_), *H*_O_, *H*_S_. To determine average pair-wise between populations (*F*_ST_) we used GenAlEx v. 6.5^37^. Arlequin 3.11^38^ was used to determine *F*_IS_ and *F*_ST_ per locus.

Analysis of molecular variance (AMOVA) to partition genetic variance was analysed in Arlequin 3.11^38^. Principal co-ordinate analysis (PCoA) to analyze genetic structure by covariance standardised approach of pairwise Nei’s genetic distances was conducted in GenAlEx version 6.5^37^. The genetic relationships using neighbour-joining (NJ) was determined in PHYLIP3.69^39^ based on Nei’s genetic distance^40^. Nei’s genetic distance was calculated in MICROSATELLITE ANALYSER (MSA) v4.05^41^.The reliability of each node was tested using 1000 resamplings. FigTree v1.3.1^42^ was used to visualize and edit the tree.

In order to determine the genetic groups among populations we used Bayesian clustering method in STRUCTURE V2.3.4^43^. This analysis was run at 35 independent runs per K Value (K1–10) with a burn-in period of 100,000 iterations and 100,000 Markov chain Monte Carlo (MCMC). Structure Harvester^44^ was used to visualize the best K value based on delta K (ΔK)^45^ and maximum log likelihood L (K)^46^. We used Mantel tests^18^ to determine the pattern of isolation by distance at 1000 permutations using GenAlEx version 6.5^37^. The comparison of the ten geographic populations distance matrix was calculated according to latitude and longitude from GPS coordinates using Vincenty’s formula, http://www.movabletype.co.uk/scripts/latlong.html.

## Electronic supplementary material

Supplementary file
