# Supplementary material for: Genetic Diversity Analysis Reveals Genetic Differentiation and Strong Population Structure in Calotropis Plants
Source: Sci Rep. 2018 May 18;8:7832. doi: 10.1038/s41598-018-26275-x (PMC5959898; doi:10.1038/s41598-018-26275-x)
Supplement: Supplementary file 1 — Supplementary file [file 41598_2018_26275_MOESM1_ESM.pdf]

Supplementary File

Genetic Diversity Analysis Reveals Genetic Differentiation and Strong Population Structure in *Calotropis* plants

Nkatha G. Muriira, Alice Muchugi, Aimin Yu, Jianchu Xu*, Aizhong Liu*

Key Laboratory for Economic Plants and Biotechnology, Yunnan, Key Laboratory for Wild Plant Resources, Kunming Institute of Botany, Chinese Academy of Sciences, Lanhei Road 132, Heilongtan, Kunming 650201, Yunnan, China

liuaizhong@mail.kib.ac.cn

Jianchu Xu: j.c.xu@cgiar.org

Supplemental Table S1-S4

Supplemental Figure S1-S3

Supplementary Table S1. Information of EST-SSR markers in Genus *Calotropis*

Primer	Gene ID	SSR Motif	Primer sequence (5'-3')	primer size range	Expected size (bp)	Ta	Putative proteins
**CG 8**	c22235	(CTCTT)5	F: TCGACGATAGATCGGAGCTT<br>R: GCCTAAATCCTTTTCGTCCC	185-205	199	55	E3 ubiquitinprotein ligase BAH1-like
**CG 29**	c12202	(TA)11	F: TAGAAACCCCACCACACCAA<br>R: CGAAGCTCAACCAAGAGGAG	218-228	175	58	peter Pan-like protein
**CG64**	c22794	(TGGATT)5	F:CCTAGCGACGTCAAAGGAAG<br>R:TCGTTTGGATAAGAGAGCCG	209-227	224	55	eukaryotic translation initiation factor 3 subunit J-like]
**CG68**	c23691	(GAAGG)6	F:GCGACTGCAATTTTCACAAA<br>R:ACAGTACCCGCCATTCAAAC	155-165	151	55	exocyst complex component 7-like
**CG90**	c12150	(CCACCG)5	F:AGGGAGATTAAACTTCACAGC<br>R:GTCATGAATCCACAACAACTC	172-208	165	55	uncharacterized protein LOC101256517 isoform 2]
**CG104**	c22703	(TGACGA)6	F:CCCGAACGAAATAAACAATACC<br>R:GGAAGAAACAGGTAAGCCACAG	140-164	151	55	Full=Hydroquinone glucosyltransferase;<br>AltName: Fullbutin synthase
**CG84**	c24197	(AGT)3	F:AACAACAATTCTTCGTCGTCGT<br>R:GCATGGATTTGGAAATACTGGT	125-155	134	57	uncharacterized protein LOC101268231]
**CG28**	c13903	(T)10(TTC)5	F:CTATGCCTCTCATCCCCAAA<br>R:AGCCATGGCAAACAAGAATC	186-198	190	58	unnamed protein product [Vitis vinifera]
**CG 35**	c10011	(TACA)5	F:CTTTCCTTCTCACCCACCAA<br>R:ATGTACCAATTCAACCCGGA	178-186	179	58	uncharacterized protein LOC101251273<br>[Solanum lycopersicum]
**CG71**	c27316	(GAA)5gtc(GAA)7	F:GAAAAGGTCCAAGATGGGTG<br>R:CTTCCCTGTCCCTGTACCTG	174-183	155	54	Dynamin-like 120 kDa protein, putative isoform 1
**CG83**	c23142	(GCC)5	F:CAGATCCGTTAACTTCCCTTGT<br>R:GTGTTGTTGTCGTCGTGTTTTT	140-155	153	56	Dynamin-like 120 kDa protein, putative isoform 1
**CG107**	c6932	(CCG)6	F:ATTATTCTCCCTCTCCCTCCAA<br>R:AAGAAGATGATGTGGGAAGCTG	157-163	159	56	protein BRASSINAZOLE-RESISTANT 1-like
**CG113**	c17964	(TCT)6	F:CCTAAAAGTCCACAACCCTGAT<br>R:GTCCGATCGGTTGGTTTTATT	114-123	120	57	probable serine/threonine-protein kinase
**CG114**	c11912	(AT)7	F:CACACACACAGAACACATCCAC<br>R:GACAGACACGCAAAAGCATAAA	140-154	144	58	E3 ubiquitin-protein ligase At4g11680-like isoform 1
**CG65**	c22892	(CAG)8	F:GTTACCGCCACCTAGGTCAA<br>R:GCTTCTTCTGGCAATCGTTC	152-182	183	55	uncharacterized protein LOC101245969 isoform 1
**CG133**	c24135	(CGC)	F:CCACACCTTCGTACACAGAAAA<br>R:GATCTACGGCAAGAAGTCATCC	250-271	254	62	transcription factor HB29-like
**CG26**	c23968	(TGT)8	F:TAGGAGCCTCGCTGTCTCTC<br>R:CGTCGTGGCTACTAATGGGT	125-140	138	59	protein TIME FOR COFFEE-like
**CG44**	c16557	(TG)12	F:CAGCCTCAGCCTCTTCACTT<br>R:GTTTGGCCGAGAAAACTCTG	160-210	165	59	L-ascorbate peroxidase T, chloroplastic-like isoform 2
**CG146**	c7583	(GCG)7	F:CGGTAGCCAAGTGAAAGCTTAG<br>R:CCTCACATAAGCATCCATACCA	192-210	209	60	uncharacterized protein LOC101258701
**CG163**	c6580	(GAT)6	F:CACACACACATATAGCCCTCCT<br>R:CACCACCTCCTCACAAACAAC	135-153	145	60	uncharacterized protein LOC101255405<br>[Solanum lycopersicum]

Ta-annealing temperature

Supplementary Table S2. Summary statistics of Genetic variation of 20 SSR loci for *Calotropis* samples

	All population N = 286						*C. procera* N = 171						*Calotropis gigantea* N=115					
Locus	N_A	H_S	H_O	*PIC*	F_{IS}	F_{ST}	*Na*	H_S	H_O	*PIC*	F_{IS}	F_{ST}	N_A	H_S	H_O	*PIC*	F_{IS}	F_{ST}
CG08	5	0.557	0.101	0.519	0.569	0.574	5	0.319	0.023	0.282	0.872	0.472	5	0.666	0.217	0.602	0.292	0.543
CG29	4	0.457	0.293	0.378	-0.061	0.393	4	0.344	0.357	0.307	-0.070	0.032	3	0.513	0.200	0.393	0.061	0.498
CG64	5	0.480	0.118	0.432	0.337	0.615	5	0.352	0.123	0.310	0.409	0.410	4	0.519	0.113	0.409	0.201	0.579
CG68	4	0.566	0.220	0.501	0.140	0.551	3	0.354	0.105	0.321	0.309	0.564	4	0.597	0.391	0.527	0.052	0.409
CG90	6	0.595	0.122	0.556	0.435	0.638	5	0.457	0.105	0.410	0.533	0.518	5	0.672	0.147	0.609	0.282	0.692
CG104	5	0.440	0.251	0.411	0.221	0.258	4	0.342	0.228	0.309	0.122	0.239	4	0.514	0.286	0.444	0.258	0.237
CG84	9	0.788	0.730	0.762	-0.483	0.374	8	0.699	0.836	0.661	-0.597	0.256	8	0.798	0.574	0.769	-0.365	0.408
CG28	8	0.824	0.339	0.800	0.163	0.508	6	0.728	0.456	0.671	0.116	0.286	7	0.638	0.165	0.576	0.240	0.586
CG35	3	0.522	0.021	0.421	0.781	0.813	3	0.131	0.023	0.124	0.808	0.051	3	0.067	0.017	0.065	0.585	0.064
CG71	2	0.108	0.115	0.102	-0.099	0.034	2	0.136	0.146	0.126	-0.106	0.024	2	0.067	0.069	0.065	-0.090	0.036
CG83	6	0.515	0.003	0.405	0.973	0.764	2	0.478	0.006	0.364	0.951	0.764	6	0.557	0.000	0.458	0.879	0.707
CG107	4	0.671	0.154	0.599	0.538	0.484	4	0.635	0.140	0.559	0.612	0.416	4	0.488	0.174	0.429	0.319	0.295
CG113	4	0.529	0.168	0.435	0.154	0.617	4	0.517	0.222	0.435	0.154	0.495	4	0.109	0.087	0.107	0.139	0.054
CG114	7	0.377	0.199	0.356	0.011	0.450	5	0.266	0.251	0.253	-0.115	0.159	6	0.484	0.122	0.423	-0.242	0.545
CG65	4	0.331	0.343	0.300	-0.441	0.259	4	0.379	0.421	0.335	-0.561	0.263	3	0.249	0.226	0.233	-0.084	0.193
CG133	8	0.660	0.206	0.613	0.355	0.516	7	0.545	0.216	0.503	0.361	0.385	7	0.611	0.191	0.547	0.275	0.457
CG26	5	0.424	0.241	0.388	0.046	0.396	3	0.366	0.309	0.316	0.014	0.142	4	0.462	0.139	0.404	-0.011	0.503
CG44	8	0.417	0.209	0.388	0.264	0.314	3	0.325	0.257	0.282	0.113	0.112	7	0.499	0.139	0.448	0.399	0.391
CG146	6	0.414	0.147	0.379	0.161	0.569	4	0.046	0.047	0.045	-0.052	0.032	5	0.660	0.296	0.595	0.133	0.463
CG163	6	0.387	0.238	0.353	-0.121	0.446	3	0.162	0.175	0.153	-0.339	0.191	6	0.558	0.330	0.471	0.008	0.447
Mean	5.45	0.503	0.211	0.455	0.196	0.479	4.2	0.379	0.223	0.338	0.177	0.291	4.85	0.487	0.194	0.429	0.167	0.405

Na-number of alleles; H_S-genetic diversity; H_O-expected heterozygosity; *PIC*- Polymorphic information index; F_{IS}; inbreeding coefficient ; F_{ST}-Genetic differentiation

Supplementary Table S3. Pairwise F_{ST} estimates of genetic differentiation for 10 *Calotropis* populations based on Nei's Distance.

	Baringo	Tharaka	Kibwezi	Tanzania	Mali K	Mali S	Dongchuan	Nepal	Hainan	Honghe
Baringo *(C. procera)*	0									
Tharaka *(C. procera)*	0.0716	0								
Kibwezi *(C. procera)*	0.0850	0.0200	0							
Tanzania *(C. procera)*	0.1105	0.0400	0.0420	0						
Mali K *(C. procera)*	0.3620	0.2950	0.3065	0.2221	0					
Mali S *(C. procera)*	0.2600	0.2090	0.2237	0.1371	0.0747	0				
Dongchuan *C. gigantea*	0.3130	0.2960	0.2990	0.2820	0.4076	0.358	0			
Nepal *C. gigantea*	0.2690	0.2490	0.2504	0.2000	0.2134	0.1638	0.2686	0		
Hainan *C. gigantea*	0.4910	0.4570	0.4538	0.4090	0.3400	0.3100	0.4514	0.1890	0	
Honghe *C. gigantea*	0.3680	0.3407	0.3439	0.3137	0.3867	0.3592	0.1761	0.2918	0.4815	0

Supplementary Table S4. Sampling location information *Calotropis* populations

**Population**	**Country**	**Species**	**Sample size**	**Latitude**	**Longitude**
Baringo	Kenya, Africa	*C. procera*	28	0.86209	36.02651
Tharaka	Kenya, Africa	*C. procera*	30	0.26475	37.90911
Kibwezi	Kenya, Africa	*C. procera*	29	-2.47106	38.04882
Tanzania	Tanzania, Africa	*C. procera*	30	-8.681561	36.92276
Mali_K	Mali, Africa	*C. procera*	27	-7.49591	14.38342
Mali_S	Mali, Africa	*C. procera*	27	-5.98412	14.05074
Dongchuan	China, Asia	*C. gigantea*	30	26.20441	103.10485
Nepal	Nepal, Asia	*C. gigantea*	25	27079826	85.65238
Hainan	China, Asia	*C. gigantea*	30	18.95386	106.69933
Honghe	China, Asia	*C. gigantea*	30	23.1	102.94924

Mali S-Segou; Mali K-Koulikoro.

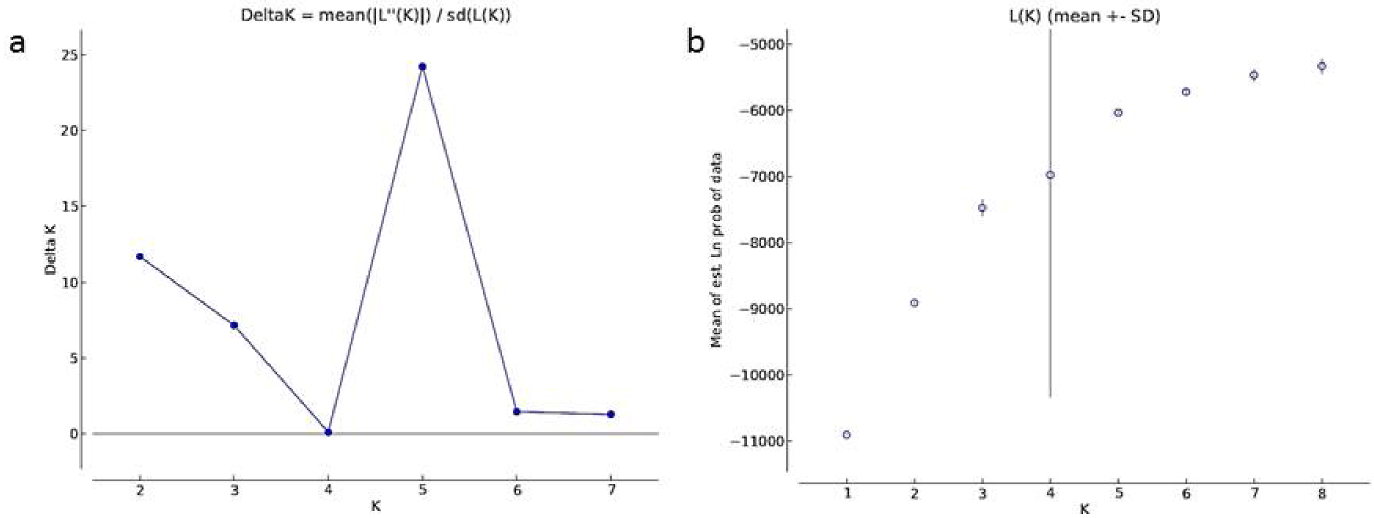

Supplementary Fig. 1. Genetic structure of 10 populations of genus *Calotropis* inferred from the admixture model (A) delta K (K = 5) and (B) model log likelihood (K = 4).

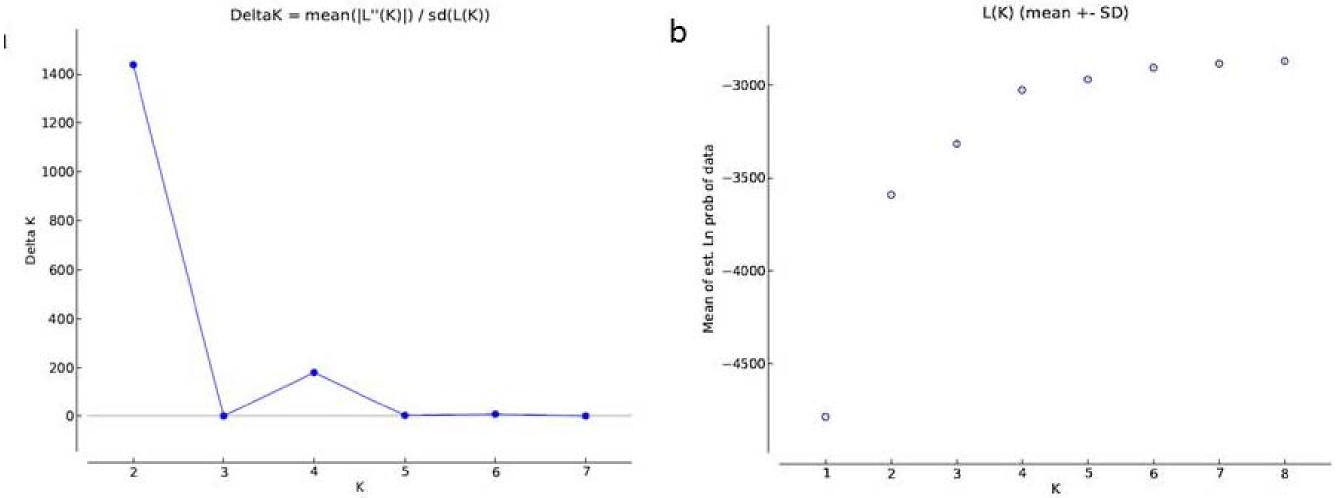

Supplementary Fig.2. Estimated ΔK (A) and L (K) (B) of 171 individuals of *Calotropis procera* from Africa over ten runs for each K value.

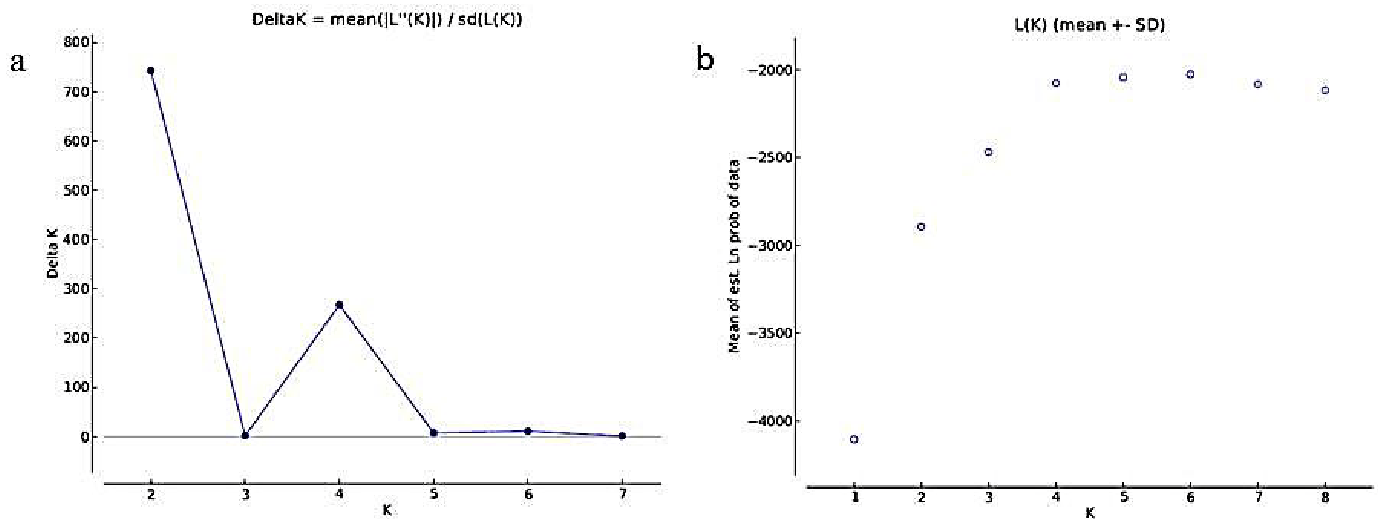

Supplementary Fig. 3. Estimated ΔK (A) and L (K) (B) of 115 individuals of *Calotropis gigantea* from Asia over ten runs for each K value.
